# A transcriptome anatomy of human colorectal cancers

**DOI:** 10.1186/1471-2407-6-40

**Published:** 2006-02-23

**Authors:** Bingjian Lü, Jing Xu, Maode Lai, Hao Zhang, Jian Chen

**Affiliations:** 1 Department of Pathology & Pathophysiology, School of Medicine, Zhejiang University, PR , 310031, China; 2 Department of Computer Science, School of Computer Science & Technology, Zhejiang University, PR, 310023, China

## Abstract

**Background:**

Accumulating databases in human genome research have enabled integrated genome-wide study on complicated diseases such as cancers. A practical approach is to mine a global transcriptome profile of disease from public database. New concepts of these diseases might emerge by landscaping this profile.

**Methods:**

In this study, we clustered human colorectal normal mucosa (N), inflammatory bowel disease (IBD), adenoma (A) and cancer (T) related expression sequence tags (EST) into UniGenes via an in-house GetUni software package and analyzed the transcriptome overview of these libraries by GOTree Machine (GOTM). Additionally, we downloaded UniGene based cDNA libraries of colon and analyzed them by Xprofiler to cross validate the efficiency of GetUni. Semi-quantitative RT-PCR was used to validate the expression of β-catenin and. 7 novel genes in colorectal cancers.

**Results:**

The efficiency of GetUni was successfully validated by Xprofiler and RT-PCR. Genes in library N, IBD and A were all found in library T. A total of 14,879 genes were identified with 2,355 of them having at least 2 transcripts. Differences in gene enrichment among these libraries were statistically significant in 50 signal transduction pathways and Pfam protein domains by GOTM analysis P < 0.01 Hypergeometric Test). Genes in two metabolic pathways, ribosome and glycolysis, were more enriched in the expression profiles of A and IBD than in N and T. Seven transmembrane receptor superfamily genes were typically abundant in cancers.

**Conclusion:**

Colorectal cancers are genetically heterogeneous. Transcription variants are common in them. Aberrations of ribosome and glycolysis pathway might be early indicators of precursor lesions in colon cancers. The electronic gene expression profile could be used to highlight the integral molecular events in colorectal cancers.

## Background

Worldwide, the incidence of colorectal cancer has been rising nowadays. It is the second mortality caused by cancers in western countries and the third or fourth in China [1]. Many tumor suppressor genes, oncogenes, and growth factor genes have been demonstrated to be involved in the carcinogenesis of colon and rectum, for example, k-ras, APC, p53 and TGFβ. A molecular genetic model of multiple genes and steps was proposed by Vogelstein in 1990 [2]. Two paralleling molecular pathways, mutator pathway and chromosomal instability pathway, were accepted since the identification of microsatellite instability (MSI) in hereditary non-polyposis colorectal cancer (HNPCC) in 1993 [3]. Four precursor lesions of colorectal cancers, adenoma, inflammatory bowel disease (ulcerous colitis and Crohn's disease), juvenile polyp and hyperplastic polyp, were mentioned in the latest edition of WHO tumour classification of digestive tract [1]. However, molecular understanding of colorectal cancer remained in its infancy. An emerging principle is that malignant transformation of colonic epithelial cells does not merely rely on mutations of generic oncogenes or tumour suppressor genes. It seems that the altered interactions among multiple genes or regulatory pathways might specifically subvert the physiological regulators of the epithelium [4]. The finished human genome was released last October in which only 20,000~25,000 genes in the human genome were estimated, far less than expected previously [5]. This also suggests that the interactions among genes or transcription level of genes really matters in both physiological and pathological processes including carcinogenesis. Hence, an integrated study on the genome-wide gene expression profile is necessary to build the corresponding gene networks underlying complicated diseases, such as colorectal cancers [6].

Experimental and computational approaches are most commonly used strategies in large-scale gene expression studies [7]. The former was represented by cDNA or serial analysis of gene expression (SAGE) library and recently developed gene expression microarray. The latter consists of various data mining methods from public database including digital differential display (DDD) in non-normalized cancer genome anatomy project (CGAP) libraries and digital Northern-blot in National Cancer Institute (NCI) SAGEMap [8,9].

Expression sequence tags (ESTs) are single passed, fragments of complementary DNA (cDNA) clones from different libraries. They are efficient for discovering novel genes in the genome [10]. It is estimated that at least 10,000 different genes are expressed in a cell with an average of about 30,000 mRNA molecules per cell. Hence, an EST library, even constructed by various modified methods such as suppression subtraction hybridization (SSH) and representational differential display (RDA), is no more than a snapshot of the mRNA composition of a certain tissue at a certain time. Particularly, genes in low-abundance might not be well represented. Fortunately, ESTs of one tissue from different libraries were available via three international nucleic acid databases, GenBank, EMBL and DDBJ. The total number of ESTs in these databases amounted to an unprecedented figure especially in the process of the initial and finished human genome sequences. Additionally, these ESTs are automatically partitioned into UniGene cluster, a non-redundant set of gene-oriented clusters [11]. Each UniGene cluster might represent one gene or at least one gene transcript. Thus, UniGene was widely applied in gene mapping, large scale gene expression, and gene alternative splicing variants identification. Therefore, it is a practical approach to mine a global transcriptome profile for a specific tissue in a certain physiological or pathological state from public databases [12].

In this study, we scrutinized the ESTs of colorectal tissues under 4 different states including normal mucosa (N), colorectal cancers (T), and two most common precancerous lesions: adenoma (A) and inflammatory bowel disease (IBD, ulcerative colitis and Crohn Disease included). Transcriptome maps of these four tissues were constructed via an in-house GetUni software package. To our knowledge, the current study is the first transcriptome map of colorectal cancers mining from NCBI EST database (dbEST) up to now. Genes in the maps were pathway oriented and analyzed by WebGestalt (WEB-based GENE SeT AnaLysis Toolkit). Several novel conceptions in colorectal tumours were emerging including heterogeneity in colorectal cancers, potential role of metabolic alterations in ribosome and glycolysis pathway in cancer precursor lesions, frequent variant transcripts in colorectal cancer as well as several novel genes in colorectal tumourigenesis, thus facilitate the understanding of molecular mechanisms underlying colorectal cancer development.

## Methods

### Resources for databases and computer programs

Human colorectal normal (N), IBD, adenoma (A) and cancer (T) associated electronic EST libraries were downloaded from dbEST [13] using keywords "(homo sapiens OR human) AND (colon OR rectal OR colorectal OR large bowel) AND normal AND gbdiv_est [PROP]", "(homo sapiens OR human) AND (colon OR rectal OR colorectal OR large bowel) AND (inflammatory bowel disease OR Crohn's disease OR ulcerative colitis) AND gbdiv_est [PROP]", "(homo sapiens OR human) AND (colon OR rectal OR colorectal OR large bowel) AND adenoma AND gbdiv_est [PROP] AND gbdiv_est [PROP]" and "(homo sapiens OR human) AND (colon OR rectal OR colorectal OR large bowel) AND (cancer OR carcinoma OR adenocarcinoma) AND gbdiv_est [PROP]", respectively. Human UniGene database (hs.data, build#181) was also downloaded from NCBI UniGene repository [14].

GetUni software package (available upon request), an in-house potent computational approach for clustering non-redundant nucleotide into UniGenes, was written in Perl6.0 and run under Linux (Redhat 9.0) operating system. The basic analytic scheme is depicted below: firstly, extract accession number from the non-redundant sequence; secondly, export related UniGene information from the downloaded UniGene database via accession number; then remove the redundant UniGene information and automatically record the hits of identical UniGene; and finally, obtain the expression profile via gene symbol for each electronic EST library.

Additionally, the cDNA libraries of colon mucosa, inflammatory bowel disease, adenoma and cancer were also downloaded from UniGene based cDNA expression profiler and analyzed by the online tool Xprofiler [15]. Genes in these libraries were applied as controls to cross validate the results from our in-house GetUni software package.

### Analysis of the transcriptome in colorectal tissues

GOTM is a web-based platform for interpreting interesting gene sets including microarray data using Gene Ontology hierarchies [16,17]. It provides classification and tissue distribution of genes as well as analytical tools (WEB-based GEne SeT AnaLysis Toolkit, WebGestalt) for comparison of the significantly enriched gene numbers in the catalogue of gene ontology in a pathway- or gene family- oriented way between an interesting and a reference gene set. A reference gene set might include all the genes in the human genome or another appropriate gene set. Hypergeometric test, appropriate for sampling without replacement, was adopted by GOTM to determine whether those enrichments are statistically significant. Since gene classification in GOTree is comprehensive and complex, we adopted genes at the default level 4 as the final export results.

### Semi-quantitative RT-PCR

In order to validate these putative genes, we selected β-catenin and 7 novel genes, which were rarely studied in colorectal cancers, for semi-quantitative RT-PCR analysis in 10 colorectal cancer tissues with matched normal mucosa and 6 colon cancer cell lines (SW620, Hce8693, Caco2, SW480, HT29, and RKO). These 7 putative genes were TWISTNB (TWIST Neighbour), SOX9 [SRY (sex determining region Y)-box 9 (campomelic dysplasia, autosomal sex-reversal)], YPEL2 [Yippee-like 2 (Drosophila)], YPEL5 [Yippee-like 5 (Drosophila)], SEPT4 (Septin 4), SEPT6 (Septin 6) and CBX3 [Chromobox homolog 3 (HP1 gamma homolog, Drosophila)]. GAPDH (Glycer aldehyde 3 phosphate dehydrogenase) was used as an internal control to normalize samples. The primers and amplification conditions of these genes are listed in Table 1. Ten microliters of PCR products were added on a 1.8% TBE agarose gel. Band intensities were measured by BioRad Quantity One Image Analysis Software. The ratio of target genes versus GAPDH in band intensity was further calculated and compared.

## Results

### Transcriptome comparison among the gene expression profiles of N, IBD, A and T

**Figure 1:**
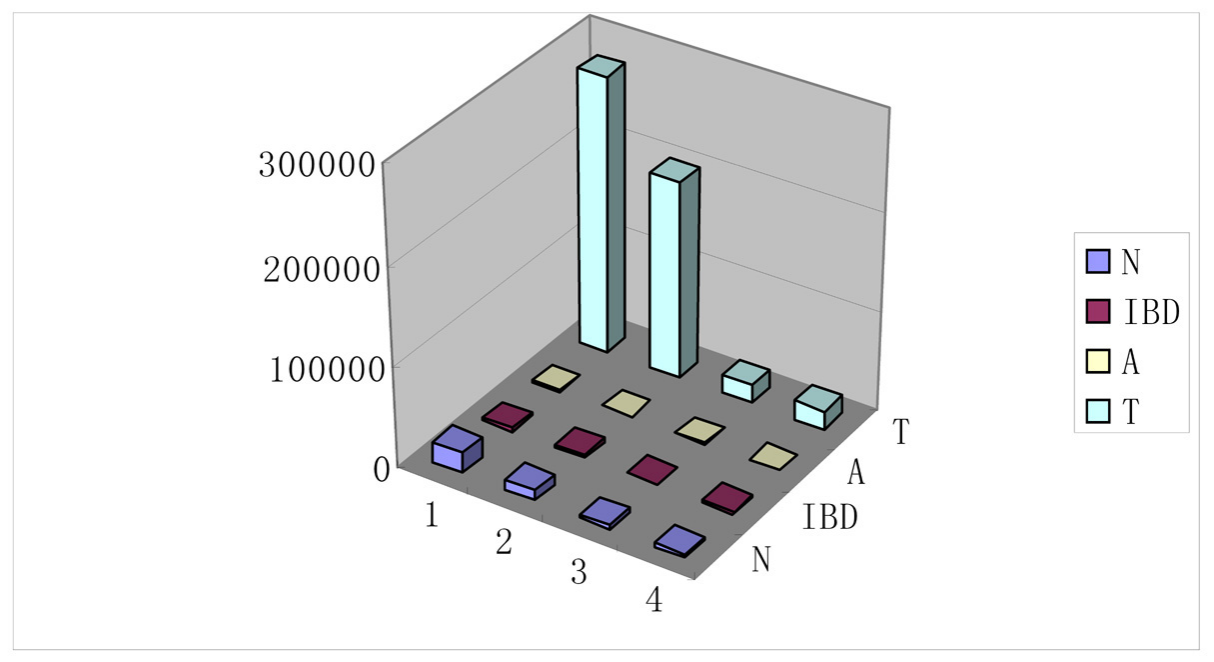
Total ESTs, UniGene transcripts, and Genes in 4 colorectal libraries. The number 1, 2, 3 and 4 in the X-axis stand for total ESTs, validated ESTs that could be clustered in to UniGene, UniGene transcripts and genes, respectively. The exact counts of each category in different states are shown in the Y-axis. Z-axis reflects different status in colon, i.e., normal (N), inflammatory bowel disease (IBD), adenoma (A) and colorectal cancer (T) as indicated by different colours in the figure. This figure implies that the increased ESTs in these libraries might be positively associated with gene redundancy in these profiles.

**Figure 2:**
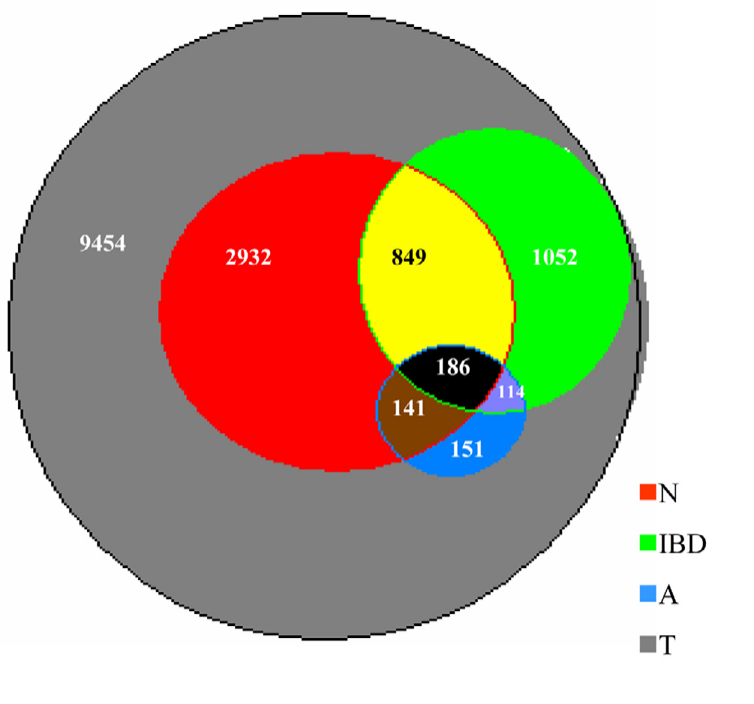
**Comparison of gene distribution in 4 gene expression profiles. **As depicted in the figure, area within the red, green, blue and grey circle is representative for the profile of N (normal), IBD (inflammatory bowel disease), A (adenoma) and T (cancer), respectively. Genes in A, IBD and N were all found in T, implying heterogeneity of colorectal cancer. Overlapping areas as filled by different colours are interpreted as the common genes among profiles. For example, the black area means the coexpressed genes in N, IBD and A, and the number "186" on it represents the total of related genes.

As shown in Figure 1, there are 20,370, 3,824, 1,270 and 279,163 ESTs in libray N, IBD, A and T, respectively. Using the in-house GetUni software, we could only cluster 10,458, 3,451, 875 and 200,608 ESTs into 4,375, 2,230, 606 and 18,891 UniGene transcripts, and eventually got 4108, 2201, 592 and 14879 genes in N, IBD, A and T libraries, respectively. ESTs which failed to be clustered into UniGene might be due to their poor quality rather than novel ESTs beyond UniGene database as we noted that most of them were genomic or mitochondria DNA fragments. The average ESTs in individual transcript were 4.66, 1.71, 2.15 and 14.78 for N, IBD, A, and T, respectively, reflecting that EST redundancy was increased with EST total sums in the library. Meanwhile, we found that 2,355 genes have at least 2 transcripts. Particularly, in the expression profile of T, the average transcripts of each gene reached to 1.27, significantly higher than in N, A and IBD (*P *< 0.01, χ^2 ^test). Surprisingly, all the genes and UniGene transcripts from A, N, and IBD libraries were found in the T library with the only exception of a transcript (Hs.329040, bA9F11.1, a hypothetical protein) which was only found in A library. Overlapping genes were common among N, IBD and A library (see Figure 2). Intriguingly, we also noted a considerable part of novel ESTs in these electronic libraries. There were 237 (5.42%, 237/4375), 65 (2.91%, 65/2230), 11 (1.82%, 11/606) and 5198 (27.52%, 5198/18891) novel ESTs in library N, IBD, A and T, respectively. A significant correlation between novel ESTs and total UniGene transcripts was revealed (p = 0.011, rho = 1, Spearman correlation).

**Figure 3:**
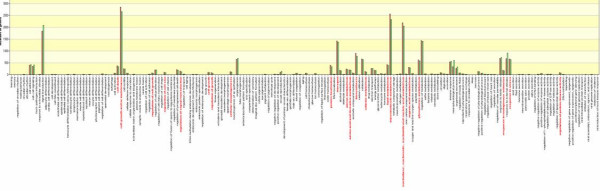
**Level 4 categories under biological_process in the T library as compared with the total genes in the whole human genome. **Red bar: genes in T (colorectal cancer) library; Blue bar: whole genes in the human genome. The different biological categories are labeled along the X-axis and the gene numbers in each catalogue are in the Y-axis. Catalogues with significantly enriched gene numbers is indicated by red words as compared with genes in the whole human genome (p < 0.01, Hypergeometric test).

GOTM analysis demonstrated that the general gene distribution was similar at level 4 in the biological processes among N, IBD, and A. Genes associated with these processes of primary metabolism, cellular metabolism, macromolecule metabolism and signal transduction were most enriched. Genes in these processes might be closely associated with the colon-specific function of differentiation. However, as for cancers, genes associated with these processes of cell growth and/or maintenance, macromolecule metabolism, nucleobase/nucleoside/nucleotide and nucleoacid metabolism and signal transduction were most frequently present (see Figure 3). In reference to the total genes of the whole human genome, genes in the biological processes as mentioned above were all significantly enriched in cancers except those in the process of signal transduction, implying the potentially pivotal and relatively specific role of these biological processes in the physiology and tumourigenesis of colon. We also classified genes in the 4 profiles of N, IBD, A, and T into 302, 269, 135 and 314 Biocarta pathways, or 131, 127, 87 and 144 KEGG pathways, or 525, 383, 178 and 1573 Pfam protein domains, respectively (See Additional file 1, 2, 3, 4). Totally, genes in 50 pathways and Pfam domains were significantly enriched in one library as compared with another (Hypergeometric Test, *P *< 0.01) (See Additional file 5). Of note, A and IBD shared enriched genes in ribosome or glycolysis pathway whereas T had enriched genes of 7 transmembrane receptor family (rhodopsin family).

### Comparison of these dbEST profiles with those of UniGene cDNA Libraries

A total of 134 cDNA libraries of colon were identified in the UniGene cDNA based expression profiler. Nevertheless, we only found 32 with definite histology diagnosis including colon mucosa 4 (i.e. normal, N), IBD 2, adenoma (A) 1 and cancer or tumour (T) 25. Using the online tool Xprofiler, we obtained 5274, 2234, 588 and 13071 UniGnene transcripts, or 5032, 2111, 566 and 11162 known genes in these UniGene library N, IBD, A and T. More than 98% genes in the UniGene based expression profile IBD, A and T were included in our corresponding libraries, respectively, in support of the potency of our GentUni software package. The minor discrepancy might reflect the updated UniGene database. We have to admit that a relatively greater gap existed between the two N profiles of the UniGene based libraries and ours since only 70% genes in UniGene N profile was identified in ours. We noticed that the annotation of these so-called normal libraries in UniGene database were "colon mucosa", not "NORMAL". Considering the stringent keywords that we used in this study, it is not strange that all these libraries were not included in our N library downloaded from the dbEST. Actually, it is inevitable that missing data could occur in any data mining studies. Fortunately, genes in the UniGene based N profile were included in our T library.

### Expression of 8 putative genes in colorectal cancers by RT-PCR

**Figure 4:**
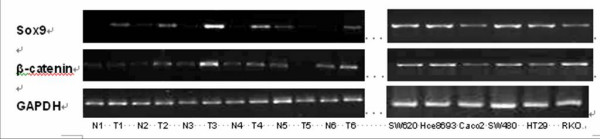
**Expression of SOX9 and β-catenin in colorectal cancers. **Semi-quantitative RT-PCR shows SOX9 and β-catenin expression in colorectal cancer tissues (Left) and cell lines (Right). Representative names of each sample or cell line are listed under the figure. T stands for cancer tissue and N for matched normal colonic mucosa. GAPDH is used as the normalization control and given at the bottom of the figure. Up-regulation of SOX9 and β-catenin is present in case 1, 2, 3, 4 and 6, and down-regulation only in 5. All cell lines have the expression of both genes.

In this electronic profile of colorectal cancer, we acquired numerous genes which were typically involved in colorectal carcinogenesis including mucin related genes, keratins, colon differentiation-related genes, tumour suppressor genes, cell cycle genes, telomerase and regulated genes, proliferation and apoptosis associated genes, etc (data not shown). More importantly, many genes, which were rarely reported in colorectal cancers, were also included. Here, we amplified 7 of such novel genes and the well-known β-catenin as a positive control. RT-PCR showed that all 8 genes were successfully amplified in colorectal tissues and cancer cells, confirming their expression in colorectal cancers, although they were not expressed in all tissues. Upregulation in cancers was seen in TWISTNB 4, YPEL2 3, CBX3 3, SOX9 8 and β-catenin 7, while downregulation was seen in TWISTNB 6, YPEL2 1, YPEL5 3, SEPT4 2, SEPT6 4, CBX3 3, SOX9 2 and β-catenin 2, compared with those in normal mucosa. The expression level in the remaining cases kept unchanged or was absent. All 6 cell lines expressed YPEL2, YPEL5, TWISTNB, CBX3, SOX9 and β-catenin. SEPT4 expression was absent in SW620 and RKO while SEPT6 expression was undetected in RKO. It was noteworthy that SOX9 was upregulated in most cancers and expressed in all 6 cell lines, in accordance with that of β-catenin (Figure 4).

## Discussion

The release of the human genome sequences and accumulating databases as well as technological development for detecting large-scale gene expression profile have enabled integrated genome-wide study on complicated pathological states, such as cancers. Tissue or organ-specific gene expression patterns as well as genome mapping have been reported via database mining including dbEST and SAGEMap [12,18]. These computational methods were proved to be effective for comparisons among tissues under different physiological or pathological states [19]. We thus developed an integrated dbEST mining procedure, GetUni. In this study, we utilized GetUni for genome-wide transcriptome analysis on normal mucosa, cancers and related precancerous lesions of colon. The efficiency of this software package was cross validated by the profiles of UniGene cDNA based libraries using the Xprofile online tool. However, we did not focus on differential gene expression patterns among these states since ESTs from dbEST were made by various methods by contributors. To our knowledge, this is the first report on computational genome-wide transcriptome analysis.

In the initial analysis of these 4 sets of transcriptome, we noticed a prominent phenomenon that all genes or gene transcripts from N, IBD and A were found in cancers. Besides the fact that the total ESTs in T were much more than those in the other 3 libraries, one of the underlying paradoxical interpretations was the complicated heterogeneity of colorectal cancers with regard to histogenesis, morphology and molecular genetics, etc. ESTs were highly redundant cDNA fragments derived from either the 3'- or 5'- regions of human genes. For example, there were 20,370 and 279,163 ESTs, and finally 4,108 and 14,879 genes in N and T library, respectively. The average ESTs per gene between T and N (18.7622, 279,163/14,879 versus 4.9586, 20,370/4,108) was approximately 5 and amounted to 7 after normalization of EST clustering efficiency (51.34% in N versus 71.86% ESTs in T which could be clustered into UniGenes, respectively). Considering that there are about 13 times more ESTs in T than in N, it is reasonable to believe that at least 50% increased ESTs in T library could be ascribed to redundancy, suggesting that increased EST was not the unique answer for the broad pattern of gene expression in cancers. Multiple pathways contributing to colorectal cancers were well established including adenoma-carcinoma sequence, inflammatory bowel disease-dysplasia-cancer, hyperplastic polyposis-intraepithelial neoplasia (IEN)-cancer or juvenile polyposis-IEN-cancer as well as direct malignant transformation from normal mucosa (*de novo*) [1]. As for morphological features, various histological subtypes with varied differentiation were fully accepted including tubular adenocarcinoma, mucinous carcinoma, signet-ring cell carcinoma, medullary carcinoma, undifferentiated carcinoma, etc. A long standing concept in cancer biology is that tumours arise and grow as a result of "tumour stem cells" or "stem cells" (multipotent progenitor cells with the capacity for self-renewal) with multiple additional mutations [20]. Consistent with this idea, a defined minority of these cells might be able to proliferate, differentiate, dedifferentiate and transdifferentiate, resulting in heterogenous gene expression patterns in cancers.

This study also found that transcript variants were quite common since 2,355 out of 14,879 genes had at least 2 transcripts. And, we believe that with the ever increasing ESTs in dbEST, more transcript variants would be discovered in these colonic tissues. In the finished human genome, a big surprise is that there are not more than 25,000 genes in the human genome, barely more than the worm *Caenorhabditis elegans *[5]. Considering the myriad cellular processes that keep our body functioning, a clear and reinforcing realization is that many genes encode more than one protein, a theory replacing the old notion of one gene one protein. One way that human genome performs such complex functions with so few genes is alternative splicing, which plays important roles in development, physiology, and disease. A genome survey of human alternative pre-mRNA splicing indicated that at least 74% of human multi-exon genes are alternatively spliced [21]. Furthermore, another intriguing phenomenon in this preliminary study is that the average transcripts of individual gene in T are higher than those in the other profiles, indicating that increased alternative splicing might be an optimal option for colorectal cancer considering their more complicated biological behaviours and functions than those of normal or benign lesions in colon tissue.

Uexpectedly, the enriched genes of ribosome in KEGG pathway were highest in 2 precancerous lesions, A and IBD, and lowest in cancer as suggested by GOTM analysis when we compared among these 4 libraries. Ribosome proteins, the major components of ribosome which is the protein synthesis center in a cell, play critical roles in physiological and pathological situations. The fundamental physiological function of colonocytes is secretion, which involves many ribosomes, as evidenced by electronic microscopy. In addition, there is highly active renewal and proliferation in crypt cells. But in cancer, this secretary capability was lost or or impaired due to dedifferentiation, resulting in the limited enrichment of ribosome genes in cancers despite that other oncogenic proteins might be actively synthesized with the absolute number of ribosome genes remaining high. This hypothesis was supported by a recent immunohistochemical study, in which 10 of 12 ribosome proteins were stained stronger in normal mucosa than in colon cancers [22]. As in adenoma and IBD, 2 intermediate states between normal mucosa and cancers, we hypothesized that the ability of secretion largely remained and that there was additional new protein synthesis to maintain the transformed phenotype in IEN or dysplasia. In our previous analysis on differentially expressed genes between colonic adenoma-normal mucosa, 6 out of 62 differentially expressed genes were ribosome proteins [23]. Ribosome protein S11 and L7 were upregulated in colonic adenomas rather than in normal mucosa or cancers as indicated by microarray [24]. An early study also demonstrated that increased mRNA levels of several ribosomal proteins were present in colorectal tumors and polyps [25]. All these findings implicated that increased synthesis of ribosomes might be an important indicator of precursor lesions of colorectal cancers.

Other findings in this study were also intriguing. Genes in the process of the KEGG Glycolysis/Gluconeogenesis pathway were significantly more enriched in adenomas and IBD than in cancers. This is consistent with a recent report on ApcMin/+ mouse model of colon tumours (26). Aberrant glucose metabolism might be emerged in the precancerous stage, earlier than before expected. Five thioredoxin family members were present in library A. These enriched genes might play important roles in anti-oxidative injury, inhibition of apoptosis, cell proliferation and differentiation [27,28]. Totally, 111 7-transmembrane receptor (rhodopsin family) superfamily members were found in cancers. Small GTP-binding protein-coupled receptors, endocrinal or neuroendocrinal receptors, and cytokine receptors were included in this catalogue. Their role in colon cancer was well documented recently [29,30].

Finally, we analyzed the expression of 8 putative genes in colorectal cancers. The preliminary data suggested that all these genes were variably expressed in cancer tissues and cell lines. Particularly, SOX9 was upregulated in most colorectal cancers. It is in consistent with our latest immunostaining results (unpublished data), indicating that SOX9 may play oncogenic roles and serve as an independent adverse prognosticator in colorectal cancers. A recent study revealed that SOX9 was an intestinal crypt specific transcription factor and downstream target of β-catenin [31]. SOX9 may thus be a potential target gene for prognostic assessment and therapeutic intervention in colorectal cancers.

## Conclusion

In this study, genome-wide transcriptome overview of colorectal cancers and their precursors were mined from dbEST using an in-house GetUni software. Landscaping the terrain of the transcriptome, new scopes in the initiation and development of colon cancers were implicated. We confirmed that colorectal cancers are genetically heterogeneous. The interactive network among these genes in colorectal cancers became more complicated due to frequent alternative splicing. Aberrations of the ribosome and glycolysis pathway might be early indicators of precursor lesions in colon cancers. Thus, the electronic gene expression profile could be used to highlight the integral molecular events in colorectal cancers. However, more effort should be put into elucidating the regulatory network among these genes via a strategy of system biology, integrating colorectal specific gene expression microarray, microfluidic card Q-PCR and proteomics study.

## Competing interests

We declare that this manuscript was not financed by any reimbursements, fees, funding, or salary from an organization except the public scientific funds as listed in the acknowledgement. No organization would gain or lose financially from the publication of this manuscript, either now or in the future. The software we designed here have been registered in China, not yet patented. Just like other universal bioinformatics tools, this software is free for anyone who is interested in his or her biomedical research. Again, we declare that no non-financial competing interests (political, personal, religious, academic, intellectual, and commercial, etc) were involved in this manuscript.

## Authors' contributions

Bingjian Lü: data analysis, co-designer of software & paper writing

Jing Xu: data collection

Maode Lai: corresponding author, principal investigator, project designation and guidance

Hao Zhang: software designer.

Jian Chen: Co-designer of software and RT-PCR analysis.

## Pre-publication history

The pre-publication history for this paper can be accessed here:



## Supplementary Material

Additional File 1 **Pathways and Pfam Prodomains in A profiles **There are 3 Excel-format tables in this file, each of which contains a cluster of KEGG pathways, Biocarta pathways, or Pfam Prodomains as indicated by the table name. In each table, there is a brief interpretation and 3 columns which stand for the pathway name, gene numbers in this pathway from the original profile and their related locus link. Each specific pathway is hyperlinked to the related website. Just double click to open these documents. Click here for file

Additional File 2 **Pathways and Pfam Prodomains in IBD profiles **The structure and usage are identical with those in Addiontonal file 1. Click here for file

Additional File 3 **Pathways and Pfam Prodomains in N profiles **The structure and usage are identical with those in Addiontonal file 1. Click here for file

Additional File 4 **Pathways and Pfam Prodomains in T profiles **The structure and usage are identical with those in Addiontonal file 1. Click here for file

Additional File 5 **Pathways or Prodomains with significantly enriched genes between 2 of 4 expression profile N, IBD, A and T **Only 1 Excel-format table is included. Double click to open this file. Pathways or pfam prodomains with significantly enriched genes were compared one library with another using Hyopergeometric test via GeneOntology analysis on line. The matched libraries were listed in Column A. For example, "IBD vs N" represents significant enriched genes in "IBD" as compared with "N". Each specific pathway is hyperlinked to the related public databases. Click here for file

## Figures and Tables

**Table 1:** Primers and amplification conditions of 8 selected putative genes

**Gene Name**	**Primers**	**PCR Fragments (bp)**	**Annealing Temperature(C)**	**Cycles**
TWISTNB	ATAACATCAAAGTTGTGGGAGA	196	48	35
	TCAGGTTTAGGAATGGAGGC			
YPEL2	ATCGAAGACTTTCCAGGCATAT	344	57	36
	ATCAGTCCCAGCCATTGTCC			
YPEL5	TAAACCTGGAGACTCGGGTGG	205	59	35
	AGCGGTTGGTCAGGATCGTAT			
Sep6	AAGGTTGCCGAACTGTCC	280	59	36
	AAGCCAACTGTGCTAACGA			
Sep4	CGAAAGTCCGTGAAGAAAG	221	59	34
	GTGTCCACAATGGTGAGCC			
CBX3	ATAATTGGTGCCACAGACA	325	53	31
	AACAACTCTTCCAACGCTA			
SOX9	AGGTGCTCAAAGGCTACGACT	360	63	32
	AGATGTGCGTCTGCTCCGTG			
β-catenin	TCTTGGCTATTACGACAG	459	58	32
	CCTCTATACCACCCACTT			
